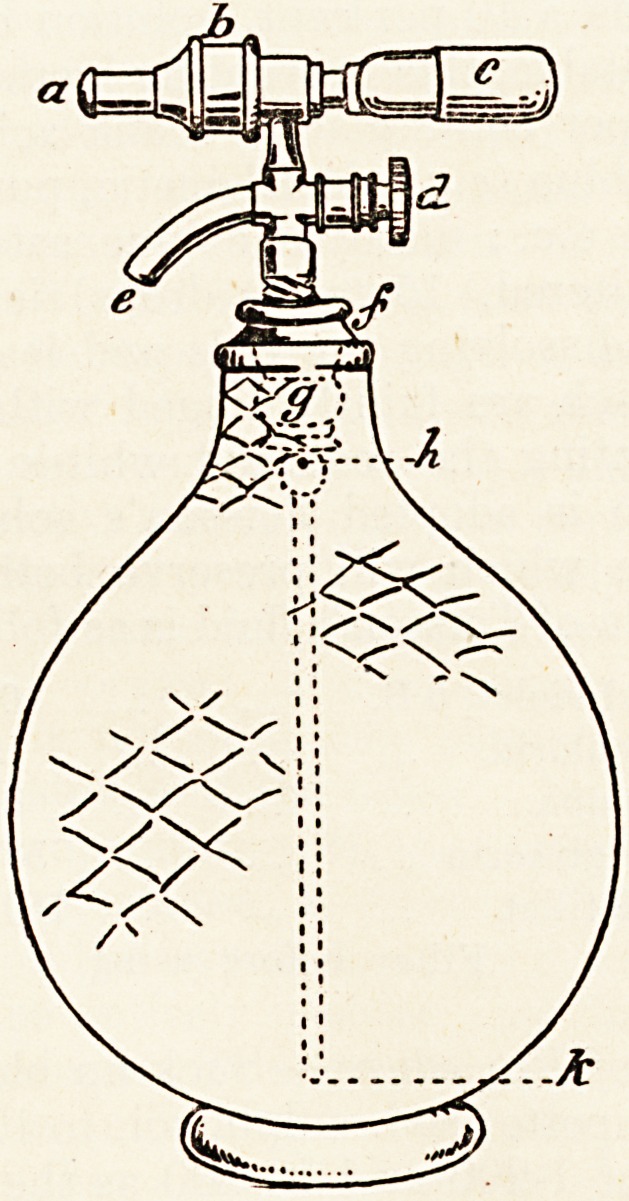# New Appliances and Things Medical

**Published:** 1910-02-05

**Authors:** 


### NEW APPLIANCES AND THINGS MEDICAL.

[We shall be glad to receive at our Office, 28 & 29 Southampton Street, Strand, London, W.C., from the manufacturers, specimens of all new preparations and appliances.]

### THE BERNARD BEER SYPHON.

The Bernard Beer Co., Ltd., Aldermary House, Watling Street, E.C., and 71 Bond Street, South Lambeth, S.W.

Most consumers prefer draught to bottled beer. Apart from purely gastronomic reasons which account for this preference, it is a well-known fact that good draught beer is superior in most respects to good bottled beer. The latter is usually pasteurised, and has its flavour altered to some extent, while beers of the heavier varieties, such as stout and porter, are apt to become acid when kept in bottle. In private houses, however, it is a matter of considerable difficulty to keep beer on draught. The casks are impossible to sterilise adequately, and the layer of air above the level of the liquid is usually not free from organisms which induce fermentation and other changes, so that the beer loses its clarity and flavour, and it becomes impossible to draw off an appetising glass with a creamy head to it.

The ingenious contrivance which the Bernard Beer Company affixes to its beer syphons seems to solve the problem of how to secure draught beer in private houses where small quantities are stored. The accompanying illustration will make its principle and application sufficiently clear. Briefly it consists of a small tube *c* of compressed $CO_2$ which is fixed to the syphon, and a most admirable arrangement for keeping a constant pressure by means of this gas upon the beer in the jar or syphon. By pressing on the spring valve *a* the gas is released and enters the syphon, keeping a constant pressure upon the level of fluid. By unscrewing *d* the beer is allowed to escape from the tap *e*. At the side there is a small rubber tube (not shown in the figure) which acts as a pressure valve. Every part of the apparatus is tested to withstand a pressure far in excess of that which it will have to bear in practice. We have given it a long and thorough trial, and are able to state that the claims made by the Company on behalf of it do not appear to us to be exaggerated. The apparatus is easily and efficiently sterilisable, and as it prevents the beer from ever coming into contact with the air (since there is always an adequate atmosphere of $CO_2$ above the sinking level of fluid) there can be no chance of aërial contamination. Beers, even of the delicate lager variety, kept in these syphons maintain their pristine excellences even after a period of several months, and the last glass drawn off is as smooth and creamy as the first. It must be distinctly understood that the $CO_2$ is not passed into the beer; the latter is never aërated or carbonated, though, owing to the plus pressure kept up in the syphon, by the liberation of the gas at the will of the drawer a small and, from a point of view of taste and flavour, entirely negligible quantity of $CO_2$ enters into solution. The syphon will not improve bad beer, but it will enable good beer to be kept practically indefinitely. It is easily manipulated, thoroughly safe, and can be honestly recommended to every private householder and every institution where draught beer is consumed. The syphons are collected by the Company—which possesses an up-to-date and specially designed cleaning and sterilising plant—sterilised, refilled, and sent back to consumers. There is thus not the slightest difficulty for those who do not wish to go to the trouble, small as it is, of cleaning the syphons themselves. The invention is a distinct advance upon any pattern we are acquainted with, and as it is hygienically sound and practically almost perfect, while moreover it fills a decided want, it should prove very popular with those who delight in a glass of honest draught beer. Full instructions are issued with every syphon, and all particulars may be obtained on application to the Company. In conclusion we may add that the filled syphons, which are admirably adapted for institutional use, are supplied at very cheap rates.

The Cancer Hospital (Free) Fulham Road, London, S.W.—The "Protean Dramatic Club" performed "The Private Secretary" for the benefit of the patients of the above hospital on Friday last. The characters of the play were excellently rendered and caused great merriment.

## Figures and Tables

**Figure f1:**